# Antimetastatic and Anti-Inflammatory Potentials of Essential Oil from Edible *Ocimum sanctum* Leaves

**DOI:** 10.1155/2014/239508

**Published:** 2014-11-05

**Authors:** Thamilvaani Manaharan, Ramaraj Thirugnanasampandan, Rajarajeswaran Jayakumar, Gunasekar Ramya, Gogul Ramnath, M. S. Kanthimathi

**Affiliations:** ^1^Department of Molecular Medicine, Faculty of Medicine, University of Malaya, 50603 Kuala Lumpur, Malaysia; ^2^Department of Biotechnology, Kongunadu Arts and Science College, GN Mills, Coimbatore, Tamil Nadu 641029, India

## Abstract

Antimetastatic and anti-inflammatory activities of *Ocimum sanctum* essential oil (OSEO) have been assessed in this study. OSEO at the concentration of 250 *μ*g/mL and above showed a significant (^*^ *P* < 0.05) decrease in the number of migrated cancer cells. In addition, OSEO at concentration of 250 *μ*g/mL and above suppressed MMP-9 activity in lipopolysaccharide (LPS) induced inflammatory cells. A dose-dependent downregulation of MMP-9 expression was observed with the treatment of OSEO compared to the control. Our findings indicate that OSEO has both antimetastatic and anti-inflammatory potentials, advocating further investigation for clinical applications in the treatment of inflammation associated cancer.

## 1. Introduction

Inflammation is one of the hallmarks of cancer initiation and progression. It contributes to tumor initiation by inducing DNA damage and chromosomal instability as well as enhancing tumor cell proliferation. Inflammation also stimulates angiogenesis and tissue remodeling, which contribute to tumor cell invasion and metastasis [1]. Many studies have shown that chronic inflammation stimulates development and progression of cancer due to the release of matrix metalloproteinases (MMPs) from the inflammatory cells [2, 3]. The MMPs function as essential regulators for the degradation of extracellular matrix (ECM) and basement membrane; thereby it contributes to the development and progression of human malignancies [4]. The MMPs can be classified into four major subgroups: collagenases (MMP-1, MMP-3, and MMP-8), gelatinases (MMP-2, MMP-9), stromelysins (MMP-3, MMP-7, MMP-10, and MMP-12), and membrane-type metalloproteinases (MTMMP-1 through MTMMP-5) [5]. The MMP-9 (92 KDa) plays a crucial role in the mechanism of tumor invasion of many types of cancer [2]. A previous report suggested a role for MMP-9 in tumor invasion relates to the fact that the release of MMP-9 is associated with the metastatic phenotype of transformed rat embryo cells [6]. Recent studies revealed that overexpression of MMP-9 in inflammation associated breast cancer [7], colon cancer [5], and ovarian cancer [8] led to tumor metastasis. Thus, inhibition of MMP-9 activity could reduce inflammation and prevent cancer progression and metastasis as well [9].

It is widely recognized that the prevention of cancer and inflammatory diseases could be associated with the intake of fresh fruits and vegetables.* Ocimum sanctum* Linn. commonly known as Tulsi or holy basil is widely known across South Asia as an aromatic medicinal herb and is distributed and cultivated worldwide [10, 11].* O. sanctum *leaves are categorized as functional foods and have a variety of pharmacological effects like antimicrobial, immunomodulatory, antistress, anti-inflammatory, antiulcer, antidiabetic, hepatoprotective, chemoprotective, hypolipidemic, cardioprotective, antioxidant, antitussive, radioprotective, memory enhancing, antiarthritic, antifertility, antihypertensive, anticoagulant, anticataract, anthelmintic, and antinociceptive effects [12–16]. The essential oil from the leaves of* O. sanctum *(OSEO), on the other hand, has been evaluated pharmacologically for antimicrobial [17], anticandidal [18], and antifungal [19, 20] activities. However, to the best of our knowledge, there is limited information about the antimetastatic and anti-inflammatory effects of OSEO. The aim of the present study was to investigate the antimetastatic and anti-inflammatory potential of OSEO using* in vitro* assays.

## 2. Materials and Methods

### 2.1. Essential Oil Extraction and Preparation

Freshly collected leaves of* O. sanctum *(1 kg) were hydrodistilled for 4 h using Clevenger apparatus for essential oil extraction. Extracted oil was treated with sodium sulphate anhydrous to remove excess water. The purified oil was then filled in small vials, tightly sealed, and stored in a refrigerator (4°C) for further studies. For the cell culture assays, stock of OSEO was prepared in dimethyl sulphoxide (1 g/mL) and was further dissolved in the culture medium (RPMI-1640).

### 2.2. Cell Migration Assay

The cell migration assay was carried out according to the method described by [21]. Photographs of three random fields were taken through an inverted microscope (Nikon, ECLIPSE TI-S), and the numbers of migrating cells were counted to calculate the average number of migrated cells from three independent experiments.

### 2.3. Anti-Inflammatory Effects of OSEO

Lipopolysaccharides (LPS) also known as lipoglycans are an endotoxin localized in the outer membrane of Gram negative bacteria (*E. coli*). In this study, LPS was used to induce inflammation [22]. Lymphocytes (1 × 10^5^ cells/well) cultured in 96 well plates using RPMI-1640 medium were induced with inflammation using LPS (1 *μ*g/mL in culture medium) for 24 h. After induction of inflammation, different concentrations (50, 100, 150, 200, and 250 *μ*g/mL) of OSEO were added to each well and incubated overnight. At the end of incubation, the cell free medium was collected and assayed for MMP-9 inhibition by gelatin zymography.

### 2.4. Gelatin Zymography

SDS-PAGE was carried out according to the method of [23]. Zymogram gel consisting of 7.5% polyacrylamide gel copolymerized with gelatin (1 mg/mL) was prepared for electrophoresis. Following electrophoresis, the gel was washed successively with 50 mL of 2.5% (v/v) Triton X-100 in distilled water for an hour to remove SDS. The gel was then incubated with developing solution (CaCl_2_ 10 mM, Triton X-100 1%, and Tris buffer, 50 mM pH 7.4) at 32°C for 18 h. Further, the gel was stained with Coomassie brilliant blue R250 for 2 h and destained overnight to reveal the bands. The bands on gel reflect the MMP-9 inhibitory effects of OSEO.

### 2.5. Reverse Transcriptase Polymerase Chain Reaction (RT-PCR Analysis)

The LPS-induced inflammatory cells were treated with different concentrations (50, 100, 150, 200, and 250 *μ*g/mL) of OSEO for 24 h. Total RNA was obtained and suspended in 9 *μ*L of deionised autoclaved DEPC treated water. Following that, 2 *μ*L of dNTP, 5 *μ*L of cDNA synthesis buffer, 1 *μ*L of oligo d (T), 1 *μ*L of reverse transcriptase enzyme mix (Thermo Fischer Scientific, India), and 9 *μ*L of nuclease free water were added and reverse transcription was carried out in a thermocycler (Eppendorf, USA) to synthesize cDNA. Primers for human MMP-9 and *β*-actin (Helini, India) were as follows; forward primer: 5′-AAG ATG CTG CTG TTC AGC GGG-3′ and reverse primer: 5′-GTC CTC AGG GCA CTG CAG GAT-3′ for MMP-9 [24] and forward primer: 5′-AGG GAA ATC GTG CGT GAC-3′ and reverse primer: 5′-CGC TCA TTG CCG ATA GTG-3′ for *β*-actin. The PCR conditions for MMP-9 and *β*-actin are as follows: initial denaturation (94°C for 5 min), denaturation (94°C for 30 sec), annealing (57°C for 30 sec for MMP-9) and (60°C for 30 sec for *β*-actin), extension (72°C for 1 min), and final extension (72°C for 10 min). The amplified fragments of MMP-9 were then loaded into the wells of 0.5% agarose gel and electrophoresis was run for 30 min at 50 volts. The gel was viewed and photographed using Gel Doc 2000 (Bio-Rad, Philadelphia, USA).

### 2.6. Statistical Analysis

Analysis at every time point from each experiment was carried out in triplicate. Means, standard errors, standard deviations, student's paired *t*-test, and one way ANOVA were calculated from replicates within the experiments and analyses were done using SPSS version 16. Statistical significance was accepted at a level of *P* < 0.05.

## 3. Results and Discussion

### 3.1. Suppression of MMP-9 and Inhibition of Cancer Cell Migration by OSEO 

**Figure 1:**
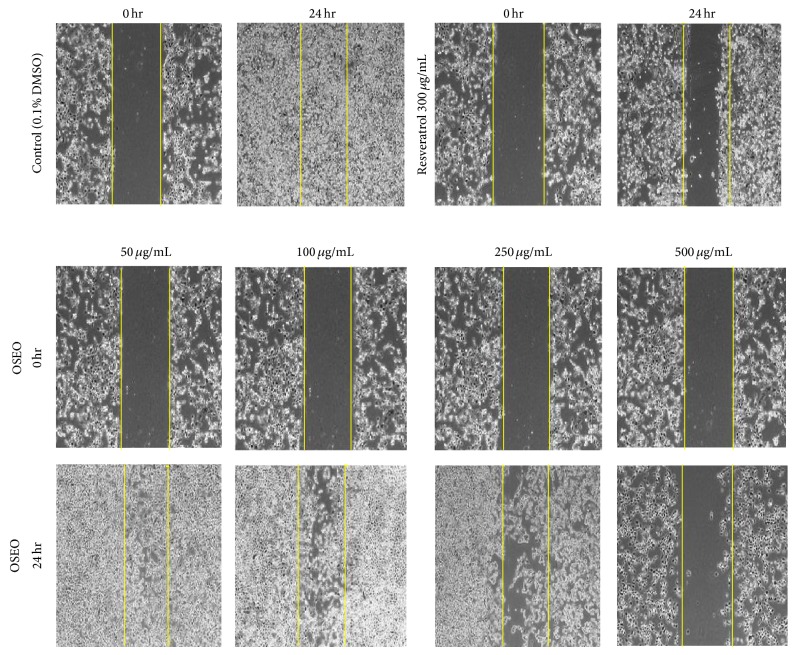
Effect of OSEO on migration of MCF-7 cells at various concentrations (50–500 *μ*g/mL). Cells treated with vehicle (0.1% DMSO) served as negative control and cells treated with resveratrol (300 *μ*g/mL) served as positive control. Photographs were captured at 0 h and 24 h of treatment on an inverted microscope (Nikon ECLIPSE TI-S) at 20x magnification. The gap or “scarring” area is marked with yellow lines.

**Figure 2:**
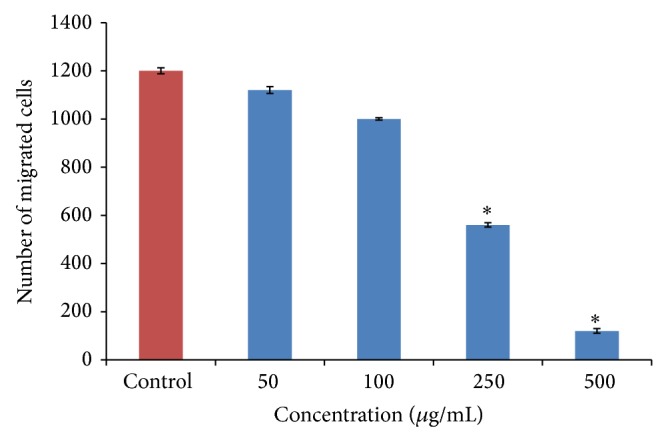
The number of migrated cells per three random fields was counted using an inverted microscope (Nikon ECLIPSE TI-S) at 400x magnification. Data are expressed as means ± SD, *n* = 3. Student's paired *t*-test showed significant values, ^*^ *P* < 0.05 versus control.

**Figure 3:**
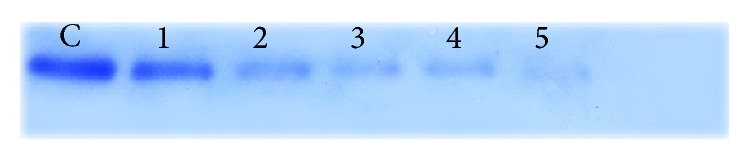
Gelatin zymography of MMP-9 activity. MMP-9 inhibition of different concentrations of OSEO after being induced with LPS. Denotation: C (Control), 1 (50 *μ*g/mL), 2 (100 *μ*g/mL), 3 (150 *μ*g/mL), 4 (200 *μ*g/mL), and 5 (250 *μ*g/mL) of OSEO. Effect of OSEO on downregulation of MMP-9.

**Figure 4:**
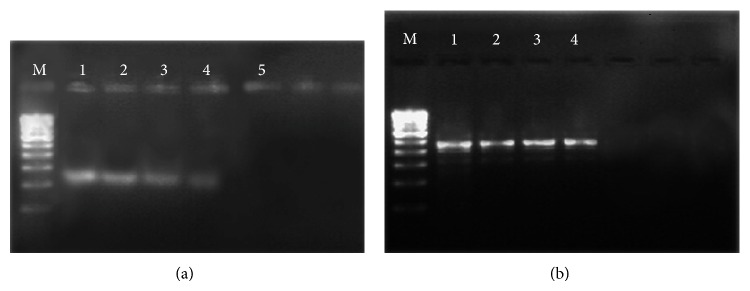
(a) Downregulation of MMP-9 was assessed after 24 h treatment with various concentrations of OSEO in LPS-induced cells. Denotation for lanes: M (100–1000 kb ladder marker), 1 (50 *μ*g/mL), 2 (100 *μ*g/mL), 3 (150 *μ*g/mL), 4 (200 *μ*g/mL), and 5 (250 *μ*g/mL) of OSEO. (b) *β*-Actin (452 bp) served as an internal marker.

Matrix metalloproteinases (MMPs), in particular, the MMP-9, are essential regulators of extracellular matrix (ECM) and recruit the inflammatory cells during chronic inflammation which involves a series of complex morphological changes in cell barrier, cell-cell interaction, and cell matrix interaction [25]. In this study, inhibition of cancer cell migration was also observed following treatment with OSEO (50–500 *μ*g/mL) (Figure 1). The OSEO at the concentration of 250 *μ*g/mL and above showed a significant (^*^ *P* < 0.05) decrease in the number of migrated cancer cells (Figure 2). In this study, we assessed the ability of OSEO as an anti-inflammatory agent to inhibit MMP-9 activity. Gelatin zymography clearly showed that OSEO inhibits MMP-9 activity in LPS-induced inflammatory cells dose-dependently compared to the control (Figure 3). No specific ladder marker was used in this study since the inhibition of MMP-9 by OSEO was compared to the control (cells treated with LPS only). Out of five different concentrations tested, 250 *μ*g/mL of oil showed the strongest MMP-9 inhibitory activity compared to the control (Figure 3). In addition, our RT-PCR analysis revealed that treatment with OSEO significantly downregulated MMP-9 expression dose-dependently compared to the LPS-induced control cells (Figure 4(a)) and the reference marker, *β*-actin (Figure 4(b)). The ladder marker 100–1000 kb was used as a reference for the amplified fragments of MMP-9 and *β*-actin genes. LPS has been recognized as the major inducer of proinflammatory cytokines, including tumor necrosis factor- (TNF-) *α* and interleukin- (IL-) 6, which in turn stimulates nitric oxide synthase (iNOS) production during the inflammatory process [26]. Thus, OSEO could also suppress the expression of proinflammatory cytokines. However, further molecular studies are required to understand its anti-inflammatory mechanism. Plant derived molecules have inhibitory effects on tumour cell migration, primarily by suppression of the activity of MMPs [27]. Our results indicate that OSEO has anti-inflammatory potential by suppressing MMP-9 expression and prevents cancer metastasis by inhibiting cancer cell migration.

## 4. Conclusion

In our study, OSEO showed strong anti-inflammatory activity by suppressing the expression of MMP-9 in LPS-induced inflammatory cells, which in turn triggered the prevention of cancer cell migration. These findings provide evidence that the antimetastatic and anti-inflammatory potential of OSEO could be useful in the development of new therapeutic strategies for inflammation associated cancer. Thus, appropriate addition of OSEO in the diet may prevent cancer and immune-mediated inflammatory diseases.

## Abbreviations

LPS: Lipopolysaccharide
MMP: Matrix metalloproteinases
OSEO: * Ocimum sanctum* essential oil.
